# Investigating the involvement of cognitive control processes in innovative and adaptive creativity and their age-related changes

**DOI:** 10.3389/fnhum.2023.1033508

**Published:** 2023-02-02

**Authors:** Boglárka Nagy, István Czigler, Petra Csizmadia, Domonkos File, Nóra Fáy, Zsófia Anna Gaál

**Affiliations:** ^1^Institute of Cognitive Neuroscience and Psychology, Research Centre for Natural Sciences, Budapest, Hungary; ^2^Department of Cognitive Science, Faculty of Natural Sciences, Budapest University of Technology and Economics, Budapest, Hungary; ^3^Institute of Psychology, Eötvös Loránd University, Budapest, Hungary; ^4^Independent Researcher, Budapest, Hungary

**Keywords:** creativity, innovative/adaptive, aging, cognitive control, task-switching, Figural TTCT, ERP

## Abstract

**Introduction:**

Based on the two-factor model of creativity, two distinct types of creative problem solving can be differentiated: innovative (“do things differently”) and adaptive (“do things better”). Flexible cognitive control is a crucial concept in connection with both general and specific styles of creativity: innovative problem-solving benefits from broader attention and flexible mental set shifting; while adaptive creativity relies on focused attention and persistent goal-oriented processes. We applied an informatively cued task-switching paradigm which is suitable for measuring different cognitive control processes and mechanisms like proactive and reactive control. We hypothesized that adaptive creativity is connected to effective proactive control processes, while innovative creativity is based on reactive task-execution. As we have found no previous evidence how age-related changes in cognitive control affects creative cognition; we also examined the effect of healthy aging on different problem-solving styles in an explorative way.

**Methods:**

Our participants, 37 younger (18–30 years) and 37 older (60–75 years) adults, were divided into innovative and adaptive creative groups according to the Torrance Test of Creative Thinking’s Figural Subtest (Hungarian version).

**Results:**

Our results showed that among younger adults the adaptively creative group had larger cue-locked CNV component (effective preparatory activity connected to proactive control), while the innovatively creative group had a larger target-locked P3b component (effective target evaluation and categorization in line with reactive control) which supports a functional difference in the two creative styles. By contrast, in older adults innovative problem-solving showed larger mixing costs (less effective maintenance and selection of task sets), and the lack of trial type effect on target-locked N2b (target-induced goal reactivation and less effective conflict resolution); while adaptive problem-solving caused them to make fewer errors (accuracy-oriented behavior).

**Discussion:**

All in all, innovative and adaptive creativity is based on distinct cognitive control mechanisms in both age-groups, but their processing level is affected by age-related changes.

## 1. Introduction

In the present study we focus on understanding which cognitive processes are necessary to be creative. As creativity is a broadly defined construct, we separated our participants based on their creative style (innovative or adaptive), as we took this to be a more relevant approach for determining what different processes may be at play in creativity. We examined in a task-switching paradigm with behavioral indicators and event-related potentials [event related potential (ERP)] which cognitive processes are involved and how they differ in creative people when compared not only with less creative ones, but also in younger and older adults.

### 1.1. Creativity

Creativity–the ability to generate and evaluate novel, appropriate, and useful ideas, or products (Guilford, 1950; Runco and Jaeger, 2012; Diedrich et al., 2015)–is crucial to the evolution of human civilization and everyday life. However, uncovering the neural and cognitive background of creative problem solving and the achievements is still largely unknown because of the complexity of this psychological construct. The challenges in creativity research arise mainly from the diverse ways used to define and measure this construct which is summarized in the New Heuristic Framework by Batey (2012). This concept integrates the level (individual, team, organization, and culture), facet (trait, process, press, and product), and measurement (objective, self-rating, and other-ratings) dimensions of creativity assessment. Moreover, it has been debated if creativity is a domain general or domain specific construct (like verbal vs. visual, artistic vs. scientific; Baer, 2010, 2015). Additionally, the creative process includes a number of different stages such as idea generation and evaluation (Wallas, 1926; Amabile, 1988; Finke et al., 1992), and these processes rely on different thinking styles. Divergent thinking is crucial for open-ended problem solving, generating novel ideas and being able to flexibly changing between them; while convergent thinking is important for closed-ended problem solving, evaluating different ideas, suppressing prepotent but irrelevant or not unique responses and selecting the most appropriate one (Guilford, 1967; Zhang et al., 2020; Cancer et al., 2022). Based on all of these different and diverse aspects and conceptualizations, there is a huge variability and lack of consensus in assessing and measuring creativity.

### 1.2. Innovative/adaptive creative style in Figural TTCT

One of the most used psychometric test to evaluate creative potential is the Torrance Test of Creative Thinking (TTCT) which is based in part on Guilford’s concept of divergent thinking and production (Guilford, 1956, 1959). It has verbal and figural subtests, and the scoring procedure has been re-normalized and re-worked many times since its first introduction in 1966 (Torrance, 1966, 1974, 1990, 1998, 2008; Torrance and Ball, 1984). The use of this creativity assessment tool has been endorsed because of its large norming samples, longitudinal validations, reasonable reliability, and high predictive validity for a wide age range for creative work, creative achievement, and creative motivation (Torrance, 1966, 1981a,1981b,^2002^; Cooper, 1991; Plucker, 1999; Cropley, 2000; Cramond et al., 2005; Kim, 2006). Additionally, the Figural TTCT subtest appears to transcend biases of gender, race, language, socioeconomic, and cultural backgrounds (Torrance, 1977; Cramond, 1993), so it can be applied generally in many different populations. Since 1984 the Figural TTCT measures the following creative thinking abilities: fluency, originality, elaboration, resistance to premature closure, abstractness of titles, and creative strengths. Originally, Torrance suggested that the different subscores represent separate dimensions of creativity. However, studies–exploring the construct validity and factor structure of the Figural TTCT–have argued whether creativity is unidimensional or multidimensional, using different factor analyses methods (confirmatory or exploratory factor analysis). Earlier research suggested that TTCT measures a general factor based on the high correlation between different subscales (mainly between fluency and originality; Dixon, 1979; Hocevar, 1979; Heausler and Thompson, 1988; Clapham, 1998). However, more recent studies have revealed that it to be more likely that TTCT captures two factors connected to creativity which are the innovative and adaptive factors (two-dimensional models of creativity; Kim, 2006; Kim et al., 2006; Krumm et al., 2014, 2016; Bart et al., 2017; Humble et al., 2018). The innovative factor includes fluency and originality; the adaptive factor contains elaboration, abstractness of titles and creative strengths (in some models the latter variable is excluded because of its different scoring and scaling nature); while resistance to premature closure loads on both factors (Kim, 2006; Kim et al., 2006) or on the adaptive one (Krumm et al., 2014, 2016).

The above concept is consistent with Kirton’s Adaption-Innovation Theory (Kirton, 1976, 1978, 2004) which suggests that individuals have a preferred approach to creative style, problem solving, as well as decision making on the innovative-adaptive spectrum (Puccio et al., 1995). Innovators aim to “do things differently,” challenge and go outside of the current and prevailing system and paradigm, and they are better in the divergent phase of problem-solving by generating many diverse and original options; while adaptors tend to “do things better,” preferring to work within the current system and paradigm while improving it by being more effective in the convergent phase of problem-solving–in selecting, evaluating, and precising options. Moreover, innovative creativity is connected to more radical, original, abstract, and problem-driven ideas, intrinsic motivation, promotion focus, and explorative behavior; while adaptive creativity is based on: more incremental, useful, concrete, and solution-driven ideas, extrinsic motivation, prevention focus, and exploitative behavior (Gilson and Madjar, 2011; Wang et al., 2021). Overall on the Figural TTCT, innovators tend to give many quick and original responses, while adaptors provide more detailed ones.

### 1.3. Flexible cognitive control, proactive/reactive control

Since creativity is a very complex construct, it can be better understood if we study it in its smaller elements. Based on the above, a possible direction is the separation along the innovative-adaptive dimensions. Our purpose in this study was to identify those cognitive processes and connected neural correlates which could be crucial for supporting creative potential, cognition, and performance, as well as showing changes regarding creative styles.

Specifically, we studied flexibility vs. persistence, and proactive vs. reactive control in a task-switching paradigm. Previous studies have established the neurocognitive framework of creativity revealing the importance of flexible cognitive control, or dynamic change and balance between flexibility and persistence in creative cognition (e.g., the dual pathway to creativity model, Nijstad et al., 2010; neurocognitive framework of the metacontrol of creative cognition, Zhang et al., 2020; for real-life creative achievement: Model of Creativity and Attention, Zabelina, 2018). Consequently, different stages, styles, attributes, and assessment of the creative cognitive process are supported by different mechanisms. On the one hand divergent thinking, idea generation, and originality (supporting innovative creative style, and real-life creative achievements) benefit more from flexibility, less effective cognitive control and shifting one’s mental set. On the other hand, convergent thinking, idea evaluation and appropriateness (connected to efficient adaptive creative style, and performance on laboratory creativity tests) benefit more from persistence, more focused cognitive control and updating one’s mental set (Nijstad et al., 2010; Runco and Jaeger, 2012; Zabelina, 2018; Kleinmintz et al., 2019; Zabelina et al., 2019; Zhang et al., 2020).

Meanwhile, cognitive control processes, the execution of goal-directed behavior based on internal or external demands (Diamond, 2013), can be applied through one of two mechanisms, either proactive or reactive control, as proposed by the dual mechanism of control framework by Braver (2012). Proactive control is the early selection stage, an ability to maintain and sustain goal-relevant information in the working memory in an anticipatory and predictive manner. So perception and attention can be shifted, action selection can be prepared, and interference and cognitive conflict can also be prevented prior to the occurrence of the cognitively demanding event. By contrast, reactive control is the late correction mechanism used in the transient stimulus-driven goal-reactivation during decision making, where attentional, interference and conflict resolution demands are recruited just after the occurrence of the target stimuli or event. For effective and successful goal-oriented behavior, both control strategies should be used efficiently but bias toward one or other of the control mechanisms, and the weighting between them can be detected at different levels: intra-individual (e.g., based on cognitive load; Speer et al., 2003); inter-individual (e.g., cognitive capacity and anxiety; Kane and Engle, 2002; Fales et al., 2008) and between populations (Braver et al., 2001). However, there is a trade-off between these two control mechanisms. While proactive control is suitable for facilitating performance, like decreasing reaction time and errors with continuous goal maintenance, it is resource intensive for the cognitive and neural system. Conversely, reactive control is more computationally efficient, but it is more sensitive to distraction because of the repeated reactivation of task goals (Braver, 2012).

The summarization of processes and concepts which could support innovative or adaptive creative style can be seen in Table 1.

**TABLE 1:** Definitions, concepts, and frameworks/models connected to innovative-adaptive dimension.

Concept	Innovative	Adaptive
**Creativity**		
Definition	Novel	Useful
Process stage	Idea generation	Idea evaluation
Thinking style	Divergent thinking	Convergent thinking
**Figural TTCT**		
Factor	Innovative creative style	Adaptive creative style
Variables	Fluency and originality	Elaboration, resistance to premature closure, and creative strengths
Kirton’s adaption-innovation theory	“Do things differently”, innovative problem-solving, explorative behavior, promotion focus	“Do things better”, adaptive problem-solving, exploitative behavior, prevention focus
**Cognitive control mechanism**		
Dual pathway to creativity/Metacontrol of creative cognition	Flexibility	Persistence (maintenance)
Dual mechanism of control framework	Reactive control	Proactive control

### 1.4. Task-switching

Informatively cued task-switching paradigm (where alternating tasks are conveyed by external cues: Rogers and Monsell, 1995; Meiran, 2000) is a suitable method for detecting those cognitive and neural processes which are connected to different cognitive control strategies. These measured processes are working memory (maintaining different task-sets, which are the stimulus-response rules), flexibility (the effective switching between task-sets), task-preparation (the cue contains information about the following target decision task), and inhibition (of the earlier task-set and response) (Kiesel et al., 2010). Moreover, cue-locked processes are relevant for observing proactive control, preparatory activities, and conflict prevention, while target-locked processes are important for detecting stages connected to reactive control, task-reactivation, and conflict resolution (Karayanidis and Jamadar, 2014). The two most examined behavioral variables in task-switching paradigms are the mixing costs (MC) and the switching costs (SC). MC manifest in slower responses in repeat trials using mixed-task blocks (two or more task-sets) compared to single-task blocks (one task-set). MC are calculated by the reaction time (RT) difference between these two conditions, and reflects working memory demand. SC manifest in slower responses in switch trials compared to repeat trials in mixed-task blocks, taken by the reaction time (RT) difference between these two conditions and reflects the index of flexibility (Monsell, 2003; Kiesel et al., 2010).

Furthermore, electrophysiological measures based on event-related potentials (ERPs) have been an important tool for detecting and distinguishing cognitive control strategies during task-switching performance. Based on previous studies (e.g., Karayanidis and Jamadar, 2014; Gaál and Czigler, 2015; Jamadar et al., 2015) we focused on the cue-locked CNV, the target-locked N2b, and the target-locked P3b components. The cue-locked ERP components reveal the proactive control processes behind task preparation. Contingent negative variation (CNV) is a negatively shifting component observable before the target-presentation at fronto-central electrode sites and reveals anticipatory activity evoked by cognitive and motor preparation in the current task (Walter et al., 1964). Its amplitude is larger for more efficient preparation (Lavric et al., 2008), evidenced in repeat trials compared to switch trials (Kieffaber and Hetrick, 2005; Nicholson et al., 2005). Therefore, the CNV allows for the indexing of better performance and anticipatory control. The examination of target-locked components in task-switching reveals the efficiency of task implementation and the required cognitive processes for reactive control. The target-locked N2b is a negative component which is evoked around 200–400 ms after the target-presentation at fronto-central electrode sites and it can reflect different cognitive control processes like the efficiency of conflict resolution and (prepotent) response inhibition (Kopp et al., 1996; Yeung and Cohen, 2006; Folstein and Van Petten, 2008). Also earlier research demonstrates there to be a larger and later N2b component in task-switching compared with task-repeat trials (Karayanidis et al., 2003, 2011; Kieffaber and Hetrick, 2005; Nicholson et al., 2005; Lavric et al., 2008; Gajewski et al., 2010; Gaál and Czigler, 2015) which accords with the interpretation that the conflict resolution and the inhibition of prepotent (previous) response makes the selection of correct response more demanding (Gajewski et al., 2018). The target-locked P3b is a very commonly examined component with parietal distribution and a maximal positive peak around 400–600 ms after the onset of target stimuli which is correlated with the allocation of cognitive resources (Gajewski et al., 2018), stimulus categorization, context and working memory updating (Donchin and Coles, 1988), response set selection (Hillyard and Kutas, 1983; Falkenstein et al., 1994), and stimulus-response link reactivation (Verleger, 2020). A smaller target-P3b amplitude is evoked in switch compared to repeat trials which is in line with the general pattern of decreased P3b amplitude for higher task demands and increased working-memory load (Karayanidis et al., 2003, 2011; Kieffaber and Hetrick, 2005).

Regarding creativity, there has been no direct comparison between creative and task-switching performance or investigation of the connection between creative styles and cognitive control strategies in task-switching. To our knowledge the only paper on this topic was about enhancing creative performance by switching between different everyday activities (Lu et al., 2017).

### 1.5. Age-related cognitive and task-switching changes

A further interest of our study was to investigate how creative style and the supporting cognitive control processes assessed with the task-switching paradigm alter with (healthy) aging. Most of the work regarding the construct validity and identifying the innovative and adaptive creative factor in the (Figural) TTCT is based on school settings (Kim, 2006; Kim et al., 2006; Krumm et al., 2014, 2016; Bart et al., 2017; Humble et al., 2018). Additionally, there are very few studies in general by any creativity assessment techniques which are investigating the changes in creative potential and style resulting from aging. The results based on the Figural TTCT and visual creativity shows contradictory age-related changes: while some studies detected age-related decline in visual creativity and figural fluency from the age 60 (Jacquish and Ripple, 1985; Palmiero et al., 2014, 2017); other studies found intact figural originality and creativity with aging (Roskos-Ewoldsen et al., 2008; Palmiero et al., 2014). However, there is no evidence how creative and problem solving style (innovative-adaptive) alters with aging.

In regards to those cognitive processes which could support the different creative styles and their age-related changes, healthy aging is accompanied by a deterioration in cognitive functions, specifically in the domains of executive functions, attention, and memory, which has already been explained by various cognitive and neurobiological theories (e.g., Drag and Bieliauskas, 2010; Goh, 2011; Grady, 2012). Among these domains, cognitive control, specifically inhibitory processes seem to have a central role in age-related cognitive decline as was first suggested by Hasher and Zacks (1988). Consequently, aging studies have usually focused on how deteriorating inhibitory functions can lead to less effective cognitive functions. Moreover, older adults showed reduced engagement of proactive control but intact reactive control, thus they relied more on reactive control (Braver et al., 2001, 2005; Jimura and Braver, 2010; Braver, 2012).

In line with the age-related changes in cognitive control strategies, Kopp et al. (2014) have found a distinct cognitive control mechanism existing between younger and older adults in task-switching. The younger group relied more on proactive control, thus on the processing of available predictive information (cue) and task anticipatory activity; while the older group, due to the lack of the relevant information selection in advance, used reactive control based on an increased activation and interference resolution during task execution. However, this bias toward reactive control in older adults supports a slower but more precise response and task execution (Czernochowski et al., 2010). In other words, while younger adults have higher cognitive flexibility and they can adjust the response criterion (the amount of evidence which is required to trigger a decision) from trial-to-trial with more flexibility, older adults are more persistent and maintain a high response criterion for all the trials (Karayanidis et al., 2011).

Investigating the age-related changes in task-switching performance and evoked ERP components (Kray and Ferdinand, 2014), mixing costs tend to be larger with age which reveals higher working memory demand for maintaining different task-sets (Karayanidis et al., 2010; Gaál and Czigler, 2015). However, switching costs show no consistent changes with aging, and is not conclusive for age-related cognitive flexibility (increased: Hillman et al., 2006; decreased: Kray, 2006, no effect of aging: Wasylyshyn et al., 2011; Gaál and Czigler, 2015). Regarding electrophysiological results, all of the commonly measured ERP components show reduction and less effective function with aging (cue-locked CNV: West and Moore, 2005; Gajewski et al., 2010; Gaál and Czigler, 2015, target-locked N2b: Friedman, 2008; Wild-Wall et al., 2008; Hämmerer et al., 2014, target-locked P3b: Gajewski et al., 2010; Karayanidis et al., 2011).

### 1.6. Hypotheses

In conclusion, for younger adults we hypothesized that more innovative people are shifting their cognitive control strategy toward reactive control and flexibility (supporting continuous shifting of mental sets, explorative behavior, reactivation of goals, and stimulus-driven decision making) which could be detected in the larger difference between switch and repeat trials in target-locked N2b and target-locked P3b, and smaller switching costs (more effective cognitive flexibility) in the applied informatively cued task-switching paradigm. For more adaptive younger adults, we assumed that their cognitive control mechanism would shift toward proactive control and persistence (supporting persistent goal-maintenance, prevention focus and desire to achieve better solutions, and to avoid errors) which could be revealed in increased cue-locked CNV and a larger difference between switch and repeat trials in this component, and as well, we predicted decreased mixing costs (increased maintenance of different task sets).

However, our study is more exploratory regarding older adults because of the lack of existing findings based on innovative and adaptive creativity in this age-group. Since there is a general shift toward reactive control and attenuation of task-switching effects in the evoked ERP components in older adults, their creative style would probably have a larger effect on their behavioral performance. Since the goal of the present study was to reveal the cognitive control processes which support different creative styles in younger and older age groups, and not age-related differences *per se*, we shall only introduce within age-group comparisons and not between age-group ones.

## 2. Materials and methods

### 2.1. Participants

This study was conducted with the participation of 43 younger (age range between 18–30 years old) and 45 older adults (between 60–75 years old). Due to the lack of evoked ERP components or extremely low number of trials after electroencephalogram (EEG) preprocessing, we excluded six younger and eight older participants’ data. Therefore, we used 37 younger (22.89 ± 2.11 years) and 37 older (67.41 ± 3.71 years) adults’ data for further analyses. In both age-groups we made a median split based on the Innovative and Adaptive Creativity Indexes separately to create less-innovative and more-innovative, as well as less-adaptive and more-adaptive groups, respectively (see details below in Section “2.4 Innovative and adaptive indexes”). The age and gender for each group can be seen in Table 2. All participants were right-handed, had normal, or corrected-to-normal vision (successful identification of Ishihara color plates, and at least 6/6 vision in a version of the Snellen charts), and had no history of any kind of neurological or psychiatric disorder.

**TABLE 2:** The demographic data of participants based on age, and assessed innovative and adaptive creativity.

	*N*	Age (years, mean ± SD)	Gender
**Younger**	**37**	**22.89 ± 2.11**	**15 males and 22 females**
Less-innovative	18	22.72 ± 1.90	5 males and 13 females
More-innovative	19	23.05 ± 2.32	10 males and 9 females
Less-adaptive	19	22.74 ± 2.08	6 males and 13 females
More-adaptive	18	23.06 ± 2.18	9 males and 9 females
**Older**	**37**	**67.41 ± 3.71**	**15 males and 22 females**
Less-innovative	18	67.17 ± 3.67	9 males and 9 females
More-innovative	19	67.63 ± 3.83	6 males and 13 females
Less-adaptive	19	68.37 ± 3.73	8 males and 11 females
More-adaptive	18	66.39 ± 3.50	7 males and 11 females

The protocol was approved by the United Ethical Review Committee for Research in Psychology [Egyesített Pszichológiai Kutatási Etikai Bizottság (EPKEB), Hungary] and the study and all of the applied methods were conducted in accordance with the Helsinki Declaration. Written informed consent was obtained from all participants, and they were paid for their contribution.

### 2.2. Procedure

Firstly, we conducted four subtests representing four major components of intelligence using the Hungarian version of the Wechsler Adult Intelligent Scale (WAIS-IV: Similarities–index of verbal comprehension, Digit Span–index of working memory, Matrix Reasoning–index of perceptual reasoning, Coding–index of processing speed, Rózsa et al., 2010); along with the updated and standardized version of the Figural Subtest of Barkóczi-Klein Creativity Test (Hungarian analog of the Circles and Incomplete figures tasks from the Torrance Test of Creative Thinking–TTCT; Barkóczi and Zétényi, 1981a,b) by Fáy et al. (2022). All participant completed these tests in order to be able to rule out those with extremely low WAIS scores as well as to measure their visual creativity.

For the experiment each participant was seated in a comfortable chair in a sound-attenuated and electrically shielded chamber. The experimental stimuli were presented with MATLAB R2015a (The MathWorks, Inc., Natick, MA, USA). using a 17-inch LCD monitor (LG Flatron L1710S, LG Electronics, Seoul, South Korea; 75Hz refresh rate) from 1.2 m distance while their EEG was recorded.

### 2.3. Task

**FIGURE 1:**
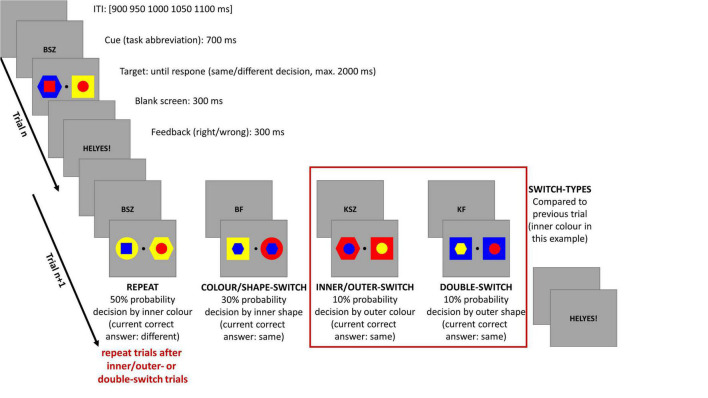
Illustration of the sequence and timing of stimuli in single and mixed trials. ITI is for intertrial interval. The red frame shows those switch-types which were analyzed in the current study. Only those repeat trials were used which were followed by an inner/outer- or double-switch trial. For a detailed description see the text above.

The experimental design is shown in Figure 1. Participants were required to undertake an informatively cued task-switching paradigm in which the actual trial’s attended attribute and task could be either the same (task-repeat and repeat trial); or else changed (task-switching and switch trial), when compared with the previous trial task.

Trials started with a letter combination cue for 700 ms which was replaced by the target stimuli until the participant made a response; or else, for a maximum of 2,000 ms. When the target was then removed, a feedback was seen on the screen for 300 ms indicating, in Hungarian, if the answer was correct or incorrect.

Target stimuli were a pair of double geometrical forms with an inner (smaller, 1.19° × 1.19° visual angle) and outer (larger, 2.38° × 2.38° visual angle) part, and a central fixation point (0.26° × 0.26° visual angle) between them. The geometrical forms were constructed from three shapes (circle, hexagon, and square) and three colors (red, blue, and yellow) in a way that the geometrical forms had to be similar in shape in one dimension (i.e., inner) and different in the other (i.e., outer); and the same method was also used for the three colors as well. The letter combinations of cues were assigned to the attended attribute of the target stimuli from which the decision and responses of the participants were then evaluated. The letter combinations contained the Hungarian abbreviations for the following: inner (**B**első); outer (**K**ülső); shape (**F**orma); and color (**SZ**ín). For example, the cue BSZ (Belső Szín–inner color) indicated that participants had to decide if the inner shapes’ color was matched or not by the target stimuli. Hand responses to shape and color, either left or right, were counterbalanced across all participants, who used a 4-button-Logitech^®^ Precision™ Gamepad (Logitech International S.A., Lausanne, Switzerland) to make their responses. If the cued attribute was the same in the two double forms, then the participants had to press the upper button on the corresponding hand’s side; or otherwise, the lower button, if the shapes or colors differed.

In the first four blocks, participants performed one of the four tasks in which inner and outer color and shape were randomly interchanged across blocks, and each of the single blocks contained 41 trials. Next, each participant performed 12 mixed blocks where the tasks were presented in a pseudorandom order, and every block contained 81 trials. In both block types, the same target could not be repeated in successive trials. The participants were shown practice blocks (for single task: 1–3 blocks, showing the four single tasks separately, based on performance, one block contained 4 × 12 trials; for mixed task: 1–3 blocks based on performance, one block contained 41 trials) before undertaking single and mixed block trials, where they could take as much time as they needed between blocks.

In the mixed blocks, the different types of task-switching conditions and their occurrence were the following: (1) repeat trials (50%, task-repeat) in which only one consecutive repeat trial was allowed; (2) color-shape or shape-color switch trials (30%, color/shape-switch, e.g., from inner color task to inner shape task); (3) inner-outer or outer-inner switch trials (10%, inner/outer-switch, e.g., from inner color task to outer color task); (4) double switch trials (10% double-switch, e.g., from inner color task to outer shape task). In the current study, we used only the inner/outer- and double-switch trials, as well as those repeat trials which followed an inner/outer- or double-switch trial. We selected these trials in order to analyze conditions with similar trial numbers that is crucial for a similar signal-to-noise ratio in EEG analysis. The two analyzed switching-types differed based on the switched dimensions, and thus, the switch-difficulty: while in inner/outer-switch trials only one attended stimuli dimension changed; whereas in double-switch trials both attended stimuli dimensions were different compared to the previous trial.

### 2.4. Innovative and adaptive indexes

We calculated variables related to figural creativity based on the method and test evaluating software from Fáy et al. (2022). The measured variables were: fluency (F: number of valid and correct answers); originality (O: calculated from the incidence of an answer matching the same answer in the database); elaboration (E: based on the minimum primary responses to the stimulus figure and the given details beyond them); resistance to premature closure (C: based on the ability to keep open and delay closure in the incomplete figures); and creative strengths (CS: 13 criterion-referenced measures about response qualities in connection with creativity^1^). The two-factor model of creativity (Kim, 2006; Kim et al., 2006; Krumm et al., 2014, 2016) was incorporated in our method, where the innovative factor is based on fluency and originality; while the adaptive factor is based on elaboration, resistance to premature closure, and creative strength. In order to reduce the high correlation between fluency and other variables (Kaufman et al., 2008; Forthmann et al., 2020), originality, elaboration, and resistance to premature closure were divided by the fluency score. Then we normalized each participant’s (fluency corrected) variable scores with the representative age group’s average scores [the updated version of the Figural Subtests of the Barkóczi-Klein Creativity Test was completed by 1,500 participants aged from 12 to 75 to create 15 representative age groups by Fáy et al. (2022)]. Finally, we calculated the Innovative and Adaptive Index separately by averaging the connected (fluency corrected andage-group normalized) variable scores from the Circles and Incomplete figures task as follow:


InnovativeIndex=[F+(Circles)F+(Incomplete_figures)O+(Circles)

O](Incomplete_figures)/4

AdaptiveIndex=[E+(Circles)E+(Incomplete_figures)

C+(Incomplete_figures)CS](all)/4

These score-normalization steps are necessary in order to calculate creativity-related indexes (Torrance, 2008) and to apply them in forming our innovative and adaptive creativity based groups. Consequently, these indexes represent the performance of each participant compared with their peers, so they are less comparable between the different age-groups.

### 2.5. Electroencephalogram recording

EEG was recorded with BrainVision Recorder 1.21.0303, ActiChamp amplifier, Ag/AgCl active electrodes, EasyCap (Brain Products GmbH, Gilching, Germany), sampling rate: 500 Hz, DC-70 Hz online filtering. We used 27 locations in accordance with the extended 10–20 system: Fp2, F7, F3, Fz, F4, F8, FC5, FC1, FC2, FC6, T7, C3, Cz, C4, T8, CP5, CP1, CP2, CP6, P7, P3, Pz, P4, P8, O1, Oz, and O2, with Fpz as ground (built-in electrode position) and the reference on the tip of the nose. Vertical eye movements (VEOG) were recorded from electrodes both above (Fp1) and below the left eyes, and horizontal eye movements (HEOG) were recorded from electrodes placed at the outer canthi of both eyes. The impedance of the electrodes was kept below 10 kΩ.

### 2.6. Electroencephalogram preprocessing

Offline EEG processing was performed in a MATLAB environment (The Mathworks, Inc.) and started with a non-causal Kaiser-windowed Finite Impulse Response filter (low pass filter parameters: 30 Hz of cut off frequency, beta of 12.2653, a transition bandwidth of 10 Hz; high pass filter parameters: 0.1 Hz of cut off frequency, beta of 5.6533, a transition bandwidth of 0.2 Hz). Independent Component Analysis (ICA) was applied on our filtered. EEG data in order to reject eye-movement artifacts (blinking, looking aside) which was performed with EEGLAB toolbox (Delorme and Makeig, 2004).

Segmentation was performed for cue-locked ERP components from −100 to 700 ms relative to the presentation of letter combination cues, and from −100 to 1000 ms relative to target stimuli presentation (a pair of double geometrical forms). Epochs were rejected from the averaging if they had larger than 100 μV voltage change between the minimum and maximum of the epoch. We used a baseline of the −100 to 0 ms interval for every trial. After EEG preprocessing, the average number of epochs in different switch-conditions were the following: repeat (192 total)–younger (mean ± SD): 147 ± 34, older: 165 ± 22; inner/outer-switch (96 total)–younger: 72 ± 17; older: 80 ± 10; double-switch (96 total)–younger: 73 ± 16; older: 81 ± 11.

### 2.7. Behavioral and ERP data analysis

We measured reaction times (RT) locked to the appearance of the target stimuli and calculated errors (percentage of all used mixed trials). Trials with incorrect responses, or reaction times which were quicker than 150 ms, were excluded from further analysis. We calculated mixing costs for every task type (inner color, inner shape, outer color, outer shape) by subtracting the given task type’s average RT in its single block from the average RT of the task’s repeat trials in mixed blocks. After that the average of the four task type’s mixing costs was taken for calculating the final mixing costs (MC). The switching costs (SC) of the different switch-types (repeat, inner/outer-switch, double-switch) were defined by finding two consecutive mixed trials where the suitable task-switching occurred from one to the next trial. These separate switching costs were calculated by subtracting the earlier trial’s RT from the later one and averaging these differences in the corresponding switch-types. Repeat benefit (RB) is the average RT decrease for a repeat trial compared to the previous switch trial.

Based on our earlier results (Gaál and Czigler, 2015, 2018), we examined cue-locked CNV, target-locked N2b, and P3b components. Every ERP component was defined by taking the average of the amplitude values of a specific time window which were the following: cue-locked CNV for 600–700 ms time window at electrode Fz and Cz; target-locked N2b for 200–350 ms time window in younger age-group and 230–380 ms time window in older age-group at electrode Fz, Cz, and Pz; target-locked P3b for 400–700 ms time window at electrode Cz and Pz.

Statistical analyses were performed with Statistica 13 (TIBCO Software Inc., Palo Alto, CA, USA). All of the analyses were performed separately for different age (younger/older) and creativity (innovative/adaptive) groups. However, we only reported those result where a significant innovative or adaptive creativity effect could be detected. All of the psychometric test scores (WAIS-IV subtests, Innovative and Adaptive Creativity Index) were compared against population means. For comparing the test scores and behavioral indexes (MC, SC, and RB) within different creativity groups (innovative/adaptive), an independent *t*-test was applied. Repeated measures of ANOVA was used for analyzing behavioral (RT, error rate) and ERP (amplitude of CNV, N2b, P3b components) data with *Innovative* (less innovative/more innovative) or *Adaptive* (less adaptive/more adaptive) as between-subject factors; and *Switch-type* (repeat, inner/outer-switch, double-switch); and *Electrode* (Fz/Cz for CNV, Fz/Cz/Pz for N2b; Cz/Pz for P3b) as within-subject factors. When comparing the difference between inner/outer- and double-switch costs, the *Switch-type* factor had different levels (inner/outer-switch, double-switch). Where Greenhouse–Geisser correction was required, p reflects the corrected values. The effect size was calculated as Cohen’s d for *t*-tests and partial eta square (η_p_^2^) for ANOVAs. *Post hoc* analysis was performed by using the Tukey’s honestly significant difference (HSD) test. In those cases where significant main effects or interactions in the ANOVA analyses were independent from the innovative/adaptive creativity (e.g., Switch-type main effect), we reported only one of the two creativity grouping factor’s results.

## 3. Results

The summary of test scores (and their comparison against population means) and behavioral data for innovative and adaptive creativity groups in our younger and older age-groups can be seen in Tables 3, 4. The summary of our results can be seen in Table 5.

**TABLE 3:** Summary of behavioral data for the more and less innovative groups (mean ± SD and *t*-test results for the difference from population average in test scores–for the WAIS-IV subtests’ scaled scores the corresponding age-group’s mean score is 10, and for the two creativity-styles’ index the corresponding age-group’s mean score is 1).

	Younger adults		Older adults	
	Less innovative	More innovative	Less innovative	More innovative
Similarities	11.11 ± 2.97	12.05 ± 2.32	14.17 ± 1.86	13.11 ± 2.51
	*t*(17) = 1.59; *p* = 0.131	*t*(18) = 3.86; *p* = 0.001	*t*(17) = 9.53; *p* < 0.001	*t*(18) = 5.38; *p* < 0.001
Digit span	10.33 ± 2.63	10.53 ± 2.41	12.89 ± 3.27	12.26 ± 3.12
	*t*(17) = 0.54; *p* = 0.598	*t*(18) = 0.95; *p* = 0.354	*t*(17) = 3.75; *p* = 0.002	*t*(18) = 3.16; *p* = 0.005
Matrix reasoning	12.06 ± 2.60	11.58 ± 2.91	12.28 ± 2.87	13.26 ± 1.94
	*t*(17) = 3.35; *p* = 0.004	*t*(18) = 2.36; *p* = 0.030	*t*(17) = 3.37; *p* = 0.004	*t*(18) = 7.34; *p* < 0.001
Coding	12.22 ± 2.02	11.84 ± 2.71	14.22 ± 2.34	15.63 ± 1.80
	*t*(17) = 4.68; *p* < 0.001	*t*(18) = 2.96; *p* = 0.008	*t*(17) = 7.65; *p* < 0.001	*t*(18) = 13.63; *p* < 0.001
Innovative index	0.81 ± 0.22	1.38 ± 0.16	1.40 ± 0.18	1.91 ± 0.26
	*t*(17) = −3.75; *p* = 0.002	*t*(18) = 10.07; *p* < 0.001	*t*(17) = 9.57; *p* < 0.001	*t*(18) = 15.10; *p* < 0.001
Adaptive index	0.95 ± 0.37	1.23 ± 0.68	1.59 ± 0.58	1.83 ± 1.37
	*t*(17) = −0.58; *p* = 0.570	*t*(18) = 1.47; *p* = 0.159	*t*(17) = 4.28; *p* < 0.001	*t*(18) = 2.62; *p* = 0.017
RT-repeat (ms)	797.51 ± 82.82	779.58 ± 161.27	970.27 ± 116.06	998.54 ± 120.77
RT-in/out (ms)	885.10 ± 109.25	871.41 ± 220.18	1104.92 ± 153.06	1105.91 ± 126.07
RT-double (ms)	907.82 ± 125.14	903.72 ± 232.60	1137.19 ± 171.86	1157.00 ± 135.58
Error-repeat (%)	8.04 ± 3.78	9.62 ± 6.35	11.55 ± 6.39	13.65 ± 6.87
Error-in/out (%)	8.62 ± 4.34	9.87 ± 4.89	11.69 ± 8.39	15.08 ± 8.72
Error-double (%)	7.06 ± 3.96	8.22 ± 6.50	9.14 ± 6.56	12.45 ± 7.52
MC (ms)	47.39 ± 59.17	64.63 ± 109.87	70.06 ± 100.42	149.56 ± 107.36
Repeat benefit (ms)	−103.07 ± 71.96	−112.40 ± 103.96	−155.59 ± 89.74	−140.06 ± 56.89
In/out SC (ms)	88.36 ± 66.89	91.77 ± 100.90	135.20 ± 80.34	116.00 ± 63.61
Double SC (ms)	101.82 ± 73.77	123.12 ± 107.51	168.79 ± 116.91	152.77 ± 70.94

The presented data from top to bottom: the assessed four subtests of the WAIS-IV; the innovative and adaptive creative style’s indexes; reaction times, and error rates for the task-switching mixed blocks’ repeat, inner/outer-, and double-switch trials; the mixing costs of the task-switching; the repeat benefit for the repeated trials; and the switching costs of inner/outer-switch and double-switch conditions in the current task-switching paradigm.

**TABLE 4:** Summary of behavioral data for the more and less adaptive groups (mean ± SD and *t*-test results for the difference from population average in test scores–for the WAIS-IV subtests’ scaled scores the corresponding age-group’s mean score is 10, and for the two creativity-styles’ index the corresponding age-group’s mean score is 1).

	Younger adults		Older adults	
	Less adaptive	More adaptive	Less adaptive	More adaptive
Similarities	10.58 ± 2.63	12.67 ± 2.30	13.26 ± 2.49	14.00 ± 1.97
	*t*(18) = 0.96; *p* = 0.350	*t*(17) = 4.92; *p* < 0.001	*t*(18) = 5.71; *p* < 0.001	*t*(17) = 8.61; *p* < 0.001
Digit span	10.05 ± 2.46	10.83 ± 2.53	12.74 ± 3.48	12.39 ± 2.89
	*t*(18) = 0.09; *p* = 0.927	*t*(17) = 1.40; *p* = 0.180	*t*(18) = 3.43; *p* = 0.003	*t*(17) = 3.50; *p* = 0.003
Matrix reasoning	11.74 ± 2.79	11.89 ± 2.76	12.26 ± 2.66	13.33 ± 2.14
	*t*(18) = 2.72; *p* = 0.014	*t*(17) = 2.90; *p* = 0.010	*t*(18) = 3.70; *p* = 0.002	*t*(17) = 6.60; *p* < 0.001
Coding	12.00 ± 2.43	12.06 ± 2.39	15.26 ± 2.00	14.61 ± 2.35
	*t*(18) = 3.59; *p* = 0.002	*t*(17) = 3.65; *p* = 0.002	*t*(18) = 11.50; *p* < 0.001	*t*(17) = 8.31; *p* < 0.001
Innovative index	1.02 ± 0.33	1.18 ± 0.34	1.71 ± 0.37	1.61 ± 0.31
	*t*(18) = 0.28; *p* = 0.779	*t*(17) = 2.28; *p* = 0.035	*t*(18) = 8.22; *p* < 0.001	*t*(17) = 8.52; *p* < 0.001
Adaptive index	0.75 ± 0.14	1.45 ± 0.62	1.24 ± 0.24	2.20 ± 1.34
	*t*(18) = −7.85; *p* < 0.001	*t*(17) = 3.09; *p* = 0.007	*t*(18) = 4.36; *p* < 0.001	*t*(17) = 3.81; *p* = 0.001
RT-repeat (ms)	803.13 ± 82.70	772.65 ± 163.78	997.86 ± 103.81	970.98 ± 132.45
RT-in/out (ms)	901.11 ± 135.58	853.75 ± 206.61	1114.60 ± 113.18	1095.75 ± 162.80
RT-double (ms)	938.11 ± 156.64	871.52 ± 211.10	1163.99 ± 128.15	1129.81 ± 176.60
Error-repeat (%)	8.22 ± 4.44	9.52 ± 6.04	14.25 ± 6.14	10.91 ± 6.86
Error-in/out (%)	8.72 ± 4.24	9.84 ± 5.03	16.34 ± 7.93	10.36 ± 8.44
Error-double (%)	7.57 ± 4.58	7.75 ± 6.23	12.88 ± 7.29	8.68 ± 6.56
MC (ms)	75.59 ± 70.77	35.82 ± 101.40	116.44 ± 82.46	105.02 ± 135.83
Repeat benefit (ms)	−121.10 ± 85.17	−93.89 ± 92.66	−144.90 ± 77.26	−150.48 ± 72.62
In/out SC (ms)	97.17 ± 73.58	82.66 ± 97.03	121.10 ± 75.87	129.82 ± 69.30
Double SC (ms)	129.64 ± 87.87	94.93 ± 95.38	168.65 ± 102.45	152.04 ± 88.73

The presented data from top to bottom: the assessed four subtests of the WAIS-IV; the innovative and adaptive creative style’s indexes; reaction times, and error rates for the task-switching mixed blocks’ repeat, inner/outer-, and double-switch trials; the mixing costs of the task-switching; the repeat benefit for the repeated trials; and the switching costs of inner/outer-switch and double-switch conditions in the current task-switching paradigm.

**TABLE 5:** Summary of our behavioral and event-related potential (ERP) results based on the applied age and creative-style grouping.

	Innovative creativity (I) in younger	Adaptive creativity (A) in younger	Innovative creativity (I) in older	Adaptive creativity (A) in older
**Behavior**				
RT	=	=	=	=
Error rate	=	=	=	more A < less A
MC	=	=	more I > less I	=
Repeat benefit	=	=	=	=
Inner/outer SC	=	=	=	=
Double SC	=	=	=	=
**ERP**				
Cue-locked CNV	=	more A > less A less A: Fz > Cz more A: Fz = Cz	=	=
Target-locked N2b	=	=	more I = less I less I: R < I/O = D more I: R = I/O = D	=
Target-locked P3b	more I > less I	=	–	–

MC, mixing costs; SC, switching costs; R, repeat; I/O, inner/outer-switch; D, double-switch. =: No significant difference between more and less innovatively/adaptively creative people in younger/older age-group.

### 3.1. Test scores

Based on the WAIS-IV subtests, our younger age-group scored similarly or higher than their peers, while our older age-group’s performance was significantly better compared to their peers (no participant had to be excluded because of low scores). Regarding creative-style grouping, the innovative and adaptive creativity indexes were suitable for significantly separating our creativity groups in both age-groups meaning in all of our four groupings more and less creative people significantly differed based on the grouping creative-style index but not based on the other one [comparing the grouping creative-style index: innovative creativity in younger: *t*(35) = 9.07, *p* < 0.001, Cohen’s *d* = 2.96; adaptive creativity in younger: *t*(35) = 4.78, *p* < 0.001, Cohen’s *d* = 1.56; innovative creativity in older: *t*(35) = 6.92, *p* < 0.001, Cohen’s *d* = 2.28; adaptive creativity in older: *t*(35) = 3.08, *p* = 0.004, Cohen’s *d* = 1.00].

### 3.2. Behavioral data

#### 3.2.1. Younger age-group

Younger age-group’s reaction time was quicker for repeated trials compared with inner/outer- (*post hoc p* < 0.001), and double switch trials (*post hoc p* < 0.001); and it was quicker for inner/outer-switch trials compared with double-switch trials at tendency level (*post hoc p* = 0.099) [*Switch-type* main effect for RT: *F*(1.42,49.77) = 42.72, *p* < 0.001, η_p_^2^ = 0.55]. Additionally, double-switch trials had a lower error rate compared to inner/outer-switch trials (*post hoc p* = 0.014), and to repeat trials at tendency level (*post hoc p* = 0.087) [*Switch-type* main effect for error rate: *F*(2,70) = 4.57, *p* = 0.014, η_p_^2^ = 0.12]. Comparing the two switch-types, double-switch trials had significantly larger switching costs compared to inner/outer-switch trials [*Switch-type* main effect for SC: *F*(1,35) = 6.71, *p* = 0.014, η_p_^2^ = 0.16].

However, behavioral variables (RT, error rate, MC, SC, RB) did not show differences based on the two creative styles (innovative, adaptive) in younger participants.

#### 3.2.2. Older age-group

In the older age-group the repeat trials showed the fastest reaction time followed by inner/outer-switch trials, and the slowest responses were given to the double-switch trials [*Switch-type* main effect for RT: *F*(2,70) = 79.47, *p* < 0.001, η_p_^2^ = 0.69; *post hoc p*-values: (repeat–inner/outer-switch) < 0.001; (repeat–double-switch) < 0.001; (inner/outer-switch–double-switch) = 0.007]. Error rates revealed that double-switch trials caused significantly less errors than inner/outer-switch trials (*post hoc p* = 0.022) [*Switch-type* main effect for error rate: *F*(1.49,52.18) = 3.84, *p* = 0.039, η_p_^2^ = 0.10]. Comparing the two switch-types, double-switch trials had significantly larger switching costs compared to inner/outer-switch trials [*Switch-type* main effect for SC: *F*(1,35) = 5.42, *p* = 0.026, η_p_^2^ = 0.13].

Looking at different creative styles, the more innovatively creative group showed larger mixing costs compared to the less innovatively creative group [*t*(35) = 2.32, *p* = 0.026, Cohen’s *d* = 0.76]. Moreover, the more adaptively creative group showed less errors than the less adaptively creative group [*Adaptive* main effect for error rates: *F*(1,35) = 4.54, *p* = 0.040, η_p_^2^ = 0.11].

### 3.3. Event-related potentials

#### 3.3.1. Cue-locked CNV

**FIGURE 2:**
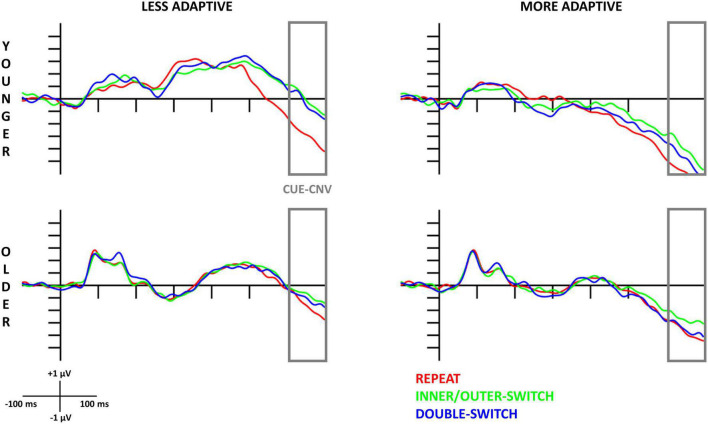
Cue-locked contingent negative variation (CNV) component at the Cz electrode site for each Switch-type in the adaptive creativity groups. The gray rectangles show the time window (600–700 ms) where the amplitude averages were calculated. The 0 ms time point on the *x*-axis is locked to the cue (letter combination) presentation.

**FIGURE 3:**
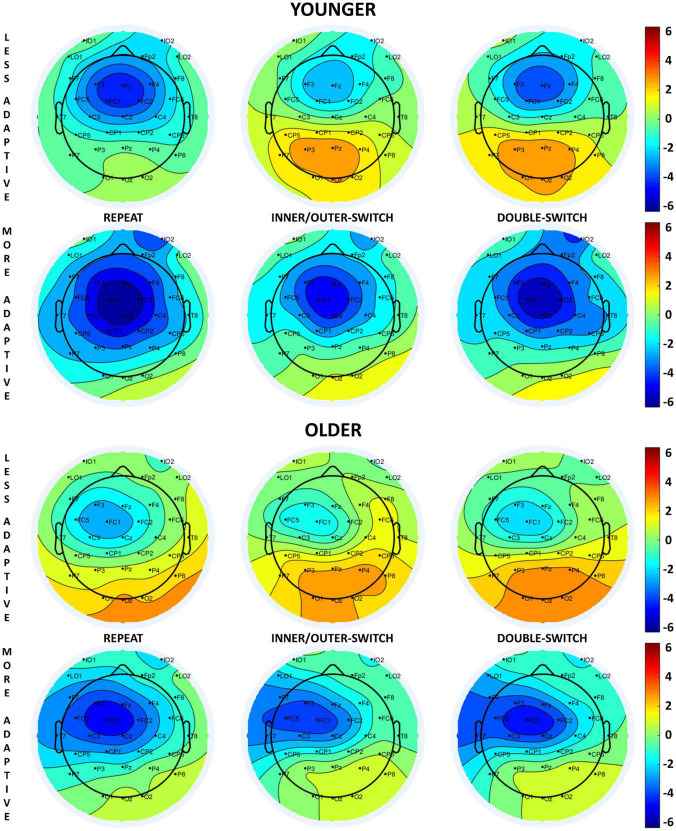
Scalp distributions of the cue-locked contingent negative variation (CNV) component at 650 ms relative to the presentation of the cue stimuli for each Switch-type in the adaptive creativity groups.

The evoked cue-locked CNV component based on adaptive creativity for younger and older age-groups can be seen in Figure 2, and the scalp distributions in Figure 3. When examining the CNV, a larger component means a larger negative amplitude.

##### 3.3.1.1. Younger age-group

Based on different switch-types, the cue-locked CNV component was the largest for repeated trials followed by double-switch trials, and the smallest component was detected for inner/outer-switch trials [*Switch-type* main effect: *F*(2,70) = 16.16, *p* < 0.001, η_p_^2^ = 0.32; *post hoc p*-values: (repeat–inner/outer-switch) < 0.001; (repeat–double-switch) = 0.002; (inner/outer-switch–double-switch) = 0.083].

The innovative creativity groups did not show any differences in the cue-locked CNV components, but there were differences between the adaptive creativity groups. Adaptively more creative younger adults had a larger CNV amplitude when compared to their less adaptively creative peers at tendency level [*Adaptive* main effect: *F*(1,35) = 3.58, *p* = 0.067, η_p_^2^ = 0.09]. In general, the CNV component was larger at Fz electrode compared to Cz [*Electrode* main effect: *F*(1,35) = 6.45, *p* = 0.016, η_p_^2^ = 0.16]. However, the *Electrode* × *Adaptive* interaction [*F*(1,35) = 9.26, *p* = 0.004, η_p_^2^ = 0.21] revealed that this electrode difference was detected just in the less adaptive group (*post hoc p* = 0.002), but not in the more adaptive group (*post hoc p* = 0.985). Additionally, this interaction showed that the more adaptive group had a larger CNV amplitude than the less adaptive group at electrode Cz (*post hoc p* = 0.045), but not at electrode Fz (*post hoc p* = 0.809).

##### 3.3.1.2. Older age-group

The *Switch-type* main effect [*F*(2,70) = 5.16, *p* = 0.008, η_p_^2^ = 0.13] showed that repeat trials evoked a larger CNV component when compared with the inner/outer-switch trials (*post hoc p* = 0.007), but the repeat–double-switch (*post hoc p* = 0.483) and inner/outer-switch–double-switch (*post hoc p* = 0.118) differences were not significant.

The different creative styles had no effect on the cue-locked CNV component in the older age-group.

#### 3.3.2. Target-locked N2b

**FIGURE 4:**
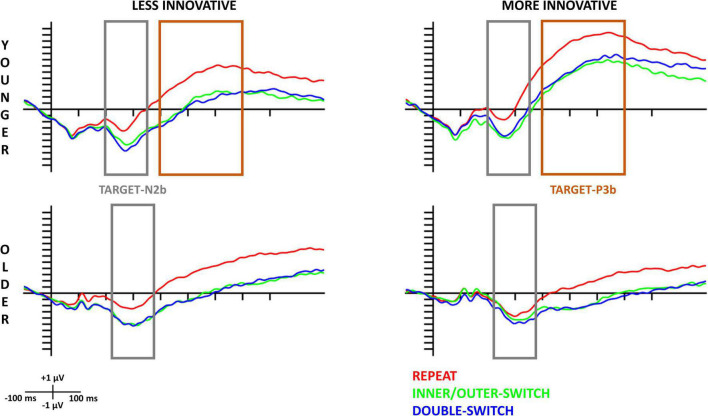
Target-locked N2b and P3b components at the Cz electrode site for each Switch-type in the innovative creativity groups. The gray rectangles show the time window (younger: 200–350 ms, older: 230–380 ms) where the amplitude averages were calculated for N2b; and the orange rectangles show the time window (400–700 ms) where the amplitude averages were calculated for P3b in the younger age-group. The 0 ms time point on the *x*-axis is locked to the target (pair of double geometrical forms) presentation.

**FIGURE 5:**
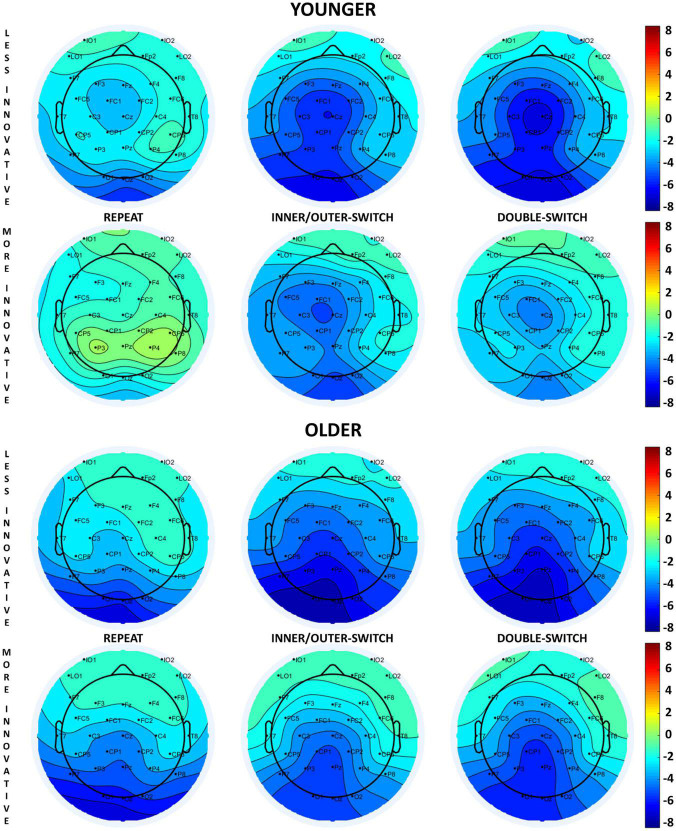
Scalp distributions of the target-locked N2b component for each Switch-type in the innovative creativity groups at: 280 ms for younger adults and 300 ms for older adults (relative to the presentation of the target stimuli).

The evoked target-locked N2b component based on innovative creativity for both age-groups can be seen in Figure 4 and the scalp distributions in Figure 5. When examining the N2b, larger component means larger negative amplitude.

##### 3.3.2.1. Younger age-group

The *Switch-type* main effect [*F*(2,70) = 30.96, *p* < 0.001, η_p_^2^ = 0.47] revealed that the target-locked N2b component was significantly smaller for repeated trials compared to inner/outer-switch (*post hoc p* < 0.001) and double-switch (*post hoc p* < 0.001) trials but the latter two did not differ from each other (*post hoc p* = 0.877). The evoked component did not show any significant difference on the three measured electrodes (Fz, Cz, Pz) [*Electrode* main effect: *F*(1.33,46.61) = 1.60, *p* = 0.215, η_p_^2^ = 0.04].

The innovative and adaptive creative style did not show any significant effects on this component in the younger age-group.

##### 3.3.2.2. Older age-group

As in the younger age-group, repeated trials evoked a smaller target-locked N2b component compared to the inner/outer-switch (*post hoc p* < 0.001) and the double-switch (*post hoc p* < 0.001) trials, but the latter two did not differ from each other (*post hoc p* = 0.672) [*Switch-type* main effect: *F*(2,70) = 20.35, *p* < 0.001, η_p_^2^ = 0.37]. Moreover, the *Electrode* main effect [*F*(1.40,48.97) = 25.83, *p* < 0.001, η_p_^2^ = 0.42] showed that the largest evoked component was at Pz electrode, followed by Cz, with the smallest being at Fz.

However, innovative creativity modified this evoked component regarding the different switch-types in the older age-group [*Switch-type* × *Innovative* interaction: *F*(2,70) = 4.19, *p* = 0.019, η_p_^2^ = 0.11]. While the less innovative group showed the same pattern as before, namely that the repeat trials evoked a smaller component compared to the inner/outer-switch and the double-switch trials [*post hoc p*-values: (repeat–inner/outer-switch) < 0.001; (repeat–double-switch) < 0.001; (inner/outer-switch–double-switch) = 1.000], in the more innovative group the three switch-type did not differ from each other based on the N2b amplitude [*post hoc p*-values: (repeat–inner/outer-switch) = 0.611; (repeat–double-switch) = 0.053; (inner/outer-switch–double-switch) = 0.771].

#### 3.3.3. Target-locked P3b

**FIGURE 6:**
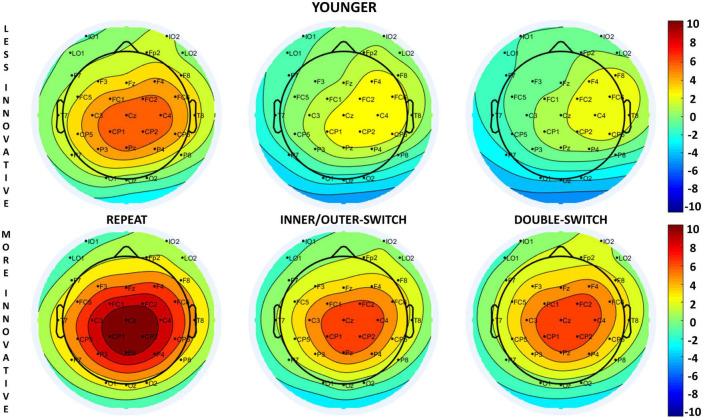
Scalp distributions of the target-locked P3b component at 600 ms (relative to the presentation of the target stimuli) for each Switch-type in the younger innovative creativity groups.

The target-locked P3b component based on innovative creativity for both age-groups can be seen in Figure 4. This figure reveals that in the older age-group we could not detect a significantly evoked P3b component for the target stimuli, thus we only analyzed this component in the younger age-group. The scalp distributions of the target-locked P3b for the younger age-group can be seen in Figure 6. When examining the P3b, a larger component means a larger positive amplitude.

##### 3.3.3.1. Younger age-group

Repeated trials showed larger target-locked P3b amplitude compared to inner/outer-switch (*post hoc p* < 0.001) and double switch (*post hoc p* < 0.001) trials, but the latter two evoked a similar P3b component (*post hoc p* = 0.994) [*Switch-type* main effect: *F*(2,70) = 38.55, *p* < 0.001, η_p_^2^ = 0.52].

This component was affected by innovative creative style in a way that the more innovative group had a larger target-locked P3b amplitude compared to the less innovative group [*Innovative* main effect: *F*(1,35) = 7.46, *p* = 0.010, η_p_^2^ = 0.18].

## 4. Discussion

### 4.1. The aim of our study

In this study we investigated how creative potential and performance can be supported by different cognitive control processes when focusing on the two creative styles: innovative and adaptive creativity. On the one hand, we were interested in cognitive control profiles based on flexibility-persistence and proactive-reactive dimensions, which could be beneficial for distinct creative problem solving styles in younger adults. On the other hand, we explored how these cognitive profiles change with healthy aging as older age-groups have not often been studied in connection with creative potential, even though there are more general and impactful cognitive changes with aging which could affect older people’s creativity. We created groups with more and less innovative and adaptive creative styles in both younger and older adults based on the Hungarian version of the figural subtests of the Torrance Test of Creative Thinking. The different cognitive control processes were defined and measured by an informatively cued task-switching paradigm which is suitable for detecting differences in flexible/persistent and proactive/reactive cognitive control dimensions using behavioral and electrophysiological (ERP) measures.

### 4.2. Cognitive control profiles in younger creativity-groups measured with task-switching

In the younger age-group participants differed in their effectiveness in proactive control regarding adaptive creativity: more adaptively creative people showed a larger and more distributed cue-locked CNV component compared to less adaptively creative people (larger cue-locked CNV was detected in processes and people relying more on proactive control in, e.g., Brunia and Van Boxtel, 2001; Lavric et al., 2008; Karayanidis et al., 2010; Karayanidis and Jamadar, 2014). However, based on innovative creativity, our younger participants showed differences in reactive control processes: the more innovative group evoked larger target-locked P3b compared to the less innovative group (larger target-locked P3b component in reactive control dominant processes and people was found in, e.g., Schmajuk et al., 2006; Gajewski and Falkenstein, 2013; Karayanidis and Jamadar, 2014). Therefore, we could find evidence for the bias toward different cognitive control mechanisms in the two creative problem solving styles in younger adults: more adaptively creative younger adults may rely more on their proactive control; whereas more innovatively creative younger adults may depend more on their reactive control. However, we were not able to detect any creativity-related differences in cognitive processes based on the flexibility-persistence dimension in our younger age-group (intact balance and efficiency in cognitive flexibility and persistence in younger adults: Karayanidis et al., 2011).

### 4.3. Cognitive control profiles in older creativity-groups measured with task-switching

Meanwhile, in the older age-group adaptive creativity showed differences in error rate: namely more adaptively creative people made less errors compared to less adaptively creative ones. This result is in line with our previous assumption that adaptive creativity could benefit from prevention focus in “doing things better.” For those with innovative creative style, more innovative older adults showed larger mixing costs compared to less innovatively creative older adults, suggesting they have a less effective working memory for maintaining different task-sets and updating their mental set. Moreover, in the more innovatively creative older group, the target-locked N2b component did not show any differences based on the different switch-types; more precisely, the inner/outer- and double-switch trials did not evoke a larger target-locked N2b compared to the repeat trials. However, both switch-types evoked larger target-locked N2b compared to repeat trials in the less innovatively creative older group (and in older groups based on adaptive creativity and all of the younger groups as well). The detected pattern in the more innovatively creative older group is in line with previous studies which found no difference between repeat and switch trials’ target-locked N2b component for older adults in task-switching paradigms (Gaál and Czigler, 2015). This reveals that conflict resolution, regarding their reactive control process, is less efficient in older innovatively creative people. At first this result seems contrary to our original hypothesis that the innovative creative style benefits from emphasized reactive control processes. However, it could be the case for older innovatively creative people that they rely more on reactive control processes, thus the transient reactivation of task sets and goals but these processes (mainly inhibitory ones) are less effective with aging. Taken together, the two ends of the creative problem solving spectrum, in our older age-group, represent two distinct cognitive aging processes. On the one hand, adaptively creative older adults showed an accuracy bias based on the speed-accuracy trade-off model for behavioral performance. This was a pattern found previously in general for older age-groups (Rabbitt, 1979; Ratcliff et al., 2007; Forstmann et al., 2011; Karayanidis et al., 2011). On the other hand, innovatively creative older adults revealed decreased function not just in persistent goal maintenance and task-set updating, shown by increased mixing costs; but also, in flexible goal reactivation and conflict resolution, revealed by the lack of switch-demand modulation on target-locked N2b component. This would be in line with the formerly popular cognitive aging framework, suggesting broad and general age-related impairments in cognitive control processes (e.g., Braver and Barch, 2002; Paxton et al., 2008; Grady, 2012); in this case in both cognitive flexibility and persistence/maintenance.

### 4.4. The common and distinct age-related patterns between creative style, problem-solving and cognitive control profile

Looking at the connection between creative and cognitive control style in both age-groups, we could detect a common pattern: innovative creativity (“do things differently,” generating many diverse and original options, related to explorative behavior) depended on target stimulus processing, evaluation and transient rule reactivation; while adaptive creativity (“do things better,” generating detailed and useful options, related to exploitative behavior) was based on facilitating accurate and effective decision-making and task-performance to the highest degree. However, this distinct task-switching execution pattern was evident at different levels in our age-groups. In the younger adults, electrophysiological measures showed differences in proactive and reactive control processes regarding adaptive and innovative creativity, respectively; though in behavioral data we could not observe any changes in the younger groups. Regarding the older adults, their behavioral data revealed more distinct patterns and differences for the two creative styles. In this age-group, whereas adaptive creativity relied on more successful cognitive aging with some compensatory mechanism; innovative creativity was evident in older people with general age-related cognitive control impairments. This age-related pattern in the (more) affected processing level of creativity could stem from a more general functional and neural processing difference between younger and older adults. In the younger age-group, not only do they have more variable neural processing and dynamics supporting constant, adaptive, and efficient behavior (McIntosh et al., 2008), but also a more stable phase- and stimulus-locked neural activity. However, in the older age-group increased intra-individual variability in behavioral and cognitive performance–like reaction time, latency of endogenous electrophysiological processes as well as increased neural noise (lower signal-to-noise ratio)–makes the analysis of ERP components less powerful (MacDonald et al., 2009).

### 4.5. Effect of creative thinking style on task difficulty

As we chose to use switch-types which differed in the number of switched dimensions in the applied task-switching paradigm, we were then interested in examining whether we could find any differences in their evaluation and execution processes; and more importantly, whether the different creative problem solving styles affected these processes in a distinguishable manner. Regarding the ERP results we could replicate earlier findings (Karayanidis and Jamadar, 2014), which revealed that repeat trials evoked a larger cue-locked CNV (Kieffaber and Hetrick, 2005; Nicholson et al., 2005) and target-locked P3b (Karayanidis et al., 2003, 2011; Kieffaber and Hetrick, 2005; Gaál and Czigler, 2015); but a smaller target-locked N2b (Karayanidis et al., 2003, 2011; Kieffaber and Hetrick, 2005; Nicholson et al., 2005; Lavric et al., 2008; Gajewski et al., 2010; Gaál and Czigler, 2015) component when compared with the switch trials. However, in the cue-locked CNV component this difference was significant only between the repeat and the inner/outer-switch trials. Examining the behavioral level, we could detect more pronounced and significant switch-type related differences: double-switch trials had larger switching costs and slower responses, but lower error rate compared to inner/outer-switch trials. Altogether, double-switch trials were more difficult to evaluate and execute as participants had to switch between inner/outer and color/shape dimensions, as well as to change simultaneously their responding hand (one hand was assigned to color decisions the other to shape decisions); we could, however, detect a bias toward accuracy mainly in this switch-type (behavioral and computational speed-accuracy trade-off: Heitz, 2014; Standage et al., 2014). Despite the participants having instruction encouraging them to respond as quickly and correctly as possible; getting feedback after every trial if it was correct or not gave them a behavioral bias toward accuracy and also to accumulate information in order to make correct responses. However, these switch-type dependent changes did not differ based on the creative style or age of the participants, so this accuracy bias was similar for each group.

### 4.6. Summary

Altogether, our results revealed distinct cognitive dynamics based on creative problem solving styles and introduced important, but so far missing evidence for the age-related changes in creative cognition. In the younger age-group adaptive and innovative creative style was supported by the bias toward proactive (larger cue-locked CNV in more adaptively creative younger adults for task-switching), and reactive (larger target-locked P3b in more innovatively creative younger adults) neural control processes, respectively. Meanwhile, in the older age-group more intact task-oriented behavior with some compensatory mechanism (lower error rate in task-switching) supported adaptive creativity. However, in this age group less effective cognitive control processes enhanced innovative creativity but impaired their task-related performance. For example larger mixing costs revealed less effective task-set maintenance and updating, and target-locked N2b showed less flexible stimulus-based task-set reactivation and impaired cognitive conflict resolution in the older innovatively creative group. Therefore, we could find specific neural and behavioral correlates which identified distinct creative problem solving styles in both younger and older adults. These results could be applied in the future for training specific cognitive control processes in order to enhance innovative or adaptive problem solving strategies as individuals can use different creative styles simultaneously for solving the same problem. Moreover, these behavioral and ERP methods can be beneficial in team working, and in creating environments to help employees to enhance their problem-solving skills depending on their innovative/adaptive styles.

### 4.7. Limitations

Even though our study brings important and novel ways of detecting cognitive mechanism behind creativity in broader age-groups, it has a few limitations. One of the main ones is, that we used cross-sectional sampling and measures to identify age-related changes in creative cognition. As a follow-up, we ought to now run additional longitudinal studies to monitor the dynamics of age-related changes in creative style and cognitive processes. Another limitation is our assessment and evaluation of creative styles, as we solely relied on the Hungarian version of the Figural TTCT to determine the innovative and adaptive creativity of the participants. Although previous factor analyses of the measured variables in Figural TTCT (e.g., Kim, 2006; Kim et al., 2006; Krumm et al., 2014, 2016) supported and determined the existence of the innovative-adaptive dimension of creativity; some further investigation is necessary to examine the existence of creative style factors using other creativity tests which capture everyday creative performance as well as connections with cognitive control processes. It is of note, that in the updated and standardized Hungarian version of the Figural TTCT (Fáy et al., 2022) and in our fluency- and age-related normalization steps there were slight differences from the original TTCT scoring system (e.g., excluding abstractness of titles, dividing variables’ score with fluency score, the calculation of age-normalization; Torrance, 2008) which makes our creativity measures not totally comparable with the results from earlier studies.

## 5. Conclusion

We could detect creative cognition differences measured with an informatively cued task-switching paradigm based on innovative-adaptive creativity factors assessed by the Hungarian version of the Figural TTCT. Moreover, we could overcome the shortcomings of creativity literature and to be able to identify age-related changes in the creative cognition profile. The younger age-group’s creative style was connected to different cognitive control style, with a bias toward proactive (for adaptive creativity) or reactive (for innovative creativity) control. While in the older age-group, a more intact cognitive profile supported their adaptive creativity, but impaired cognitive control processes and task-oriented behavior, in both flexibility and persistence/maintenance dimensions, enhanced their innovative creativity. Our results point to creativity being understood if we can study it in its smaller elements: a promising way is separating creative style and using a domain specific approach. These findings can be potentially useful for optimizing and improving creative problem solving abilities in everyday and workplace settings for both younger and older adults.

## Data availability statement

The original contributions presented in this study are publicly available. This data can be found here: https://web.gin.g-node.org/gaalzs/CREATS.

## Ethics statement

The studies involving human participants were reviewed and approved by the United Ethical Review Committee for Research in Psychology (Egyesített Pszichológiai Kutatási Etikai Bizottság, Hungary). The patients/participants provided their written informed consent to participate in this study.

## Author contributions

BN, IC, DF, and ZG designed the study. NF updated and standardized the Hungarian version of the creativity test. PC and BN evaluated the creativity tests and collected and analyzed the data. BN, ZG, and IC wrote the manuscript. All authors contributed to the article and approved the submitted version.

## Footnotes

1 Emotional expressiveness, storytelling articulateness, movement or action, expressiveness of titles, synthesis of incomplete figures, synthesis of lines, unusual visualization, internal visualization, extending or breaking boundaries, humor, richness of imagery, colorfulness of imagery, fantasy.
